# Family Context and Khat Chewing among Adult Yemeni Women: A Cross-Sectional Study

**DOI:** 10.1155/2014/505474

**Published:** 2014-05-20

**Authors:** AL-abed Ali AL-abed, Rosnah Sutan, Sami Abdo Radman Al-Dubai, Syed Mohamed Aljunid

**Affiliations:** ^1^Community Health Department, UKM Medical Centre (UKMMC), Jalan Yaacob Latiff, Cheras, 56000 Kuala Lumpur, Malaysia; ^2^United Nations University-International Institute for Global Health, UNU-IIGH Building, UKM Medical Centre (UKMMC), Jalan Yaacob Latiff, 56000 Kuala Lumpur, Malaysia; ^3^Department of Community Medicine, International Medical University (IMU) No. 126, Jln Jalil Perkasa 19, Bukit Jalil, 57000 Kuala Lumpur, Malaysia; ^4^International Centre for Case-Mix and Clinical Coding (ITCC), UKM Medical Centre (UKMMC), Jalan Yaacob Latiff, Cheras, 56000 Kuala Lumpur, Malaysia

## Abstract

Khat chewing is associated with unfavourable health outcomes and family dysfunction. Few studies have addressed the factors associated with khat chewing among Yemeni women. However, the family and husband effects on chewing khat by women have not been addressed. This study aimed to determine the prevalence of khat chewing among Yemeni women and its associated factors, particularly husbands and family factors. A cross-sectional study was conducted among 692 adult Yemeni women in the city of Sana'a in Yemen using structured “face to face” interviews. Mean (±SD) age of women was 27.3 years (±6.10). The prevalence of chewing khat by women was 29.6%. Factors associated with chewing khat among women were chewing khat by husbands (OR = 1.8; 95% CI: 1.26, 2.53), being married (OR = 2.0; 95% CI: 1.20, 3.37), frequent family social gatherings (OR = 1.5; 95% CI: 1.06, 2.10), high family income (OR = 1.57; 95% CI: 1.12, 2.21), larger house (OR = 1.63; 95% CI: 1.16, 2.31), and age of women (OR = 0.64; 95% CI: 0.44, 0.92). It is concluded that khat chewing by women in this study was significantly associated with family factors and with khat chewing by their husbands. Urgent action is needed to control khat chewing particularly among women.

## 1. Introduction

Khat is derived from the fresh leaves and buds of* Catha edulis*, which is endemic and very popular plant in Africa and Yemen [1]. Its active ingredients comprise cathinone, cathine, and norephedrine, which are analogous to amphetamine [2]. In the last few decades and due to the emigration paths, khat “habit” has spread throughout many African countries and from there to Europe, Australia, and the United States [3]. In the UK, the prevalence of khat chewing among Somalis ranges from 24.0% to 67.0% [4].

In Yemen, the prevalence of khat chewing for the entire population is 67.9%. This includes 80.0% of men and 60.0% of women. Current “daily use” is estimated at 23.6% of the general population (31.8% of men and 8.9 of women) [5]. Khat chewing deeply affects all facets of Yemeni social life and has become an essential part of social gatherings and activities [3]. Yemenis believe that khat chewing increases energy and relieves depression and physical fatigue [6].

It has been reported that khat chewing adversely affects Yemen's economic development and causes public health problems [7]. Approximately 50.0% of household income is spent daily on khat [8] and its use has been associated with unemployment, decreased family income, lower levels of education, depressed living conditions, family dysfunction, and wasting of time [4, 9–11]. Khat chewing is also associated with an increased risk of acute myocardial infarction [12], high blood pressure [13], oral cancer [14–16], and haemorrhoids [17]. Malnutrition and loss of appetite have also been observed among khat users in addition to gastritis and constipation [14, 18].

Khat chewing has recently become common among pregnant women in Yemen (approximately 41.0%) [19]. In the previous studies, khat chewing among pregnant women was associated with reduced daily food intake, anaemia, disturbance of foetal growth, low birth weight, perinatal and infant death, and other obstetric health problems [20–23].

In the past few decades, khat chewing by women was considered unacceptable, but recently its use among women and children has increased and become socially accepted [24, 25]. It was thought that husbands were responsible for these changes because they encourage their wives to share them chewing khat [26].

Few studies have addressed the factors associated with khat chewing among Yemeni women. These studies have demonstrated that khat chewing was associated with age, gender, residence, and occupation [27]. However, the family and husband effects on chewing khat by women have not been addressed in the literature. Hence, this study was designed to determine the prevalence of khat chewing and its associated factors, particularly husbands and family factors. In this study, we hypothesized that the prevalence of khat chewing is higher among women whose husbands or family chewed khat.

## 2. Materials and Methods

### 2.1. Study Design and Population

A cross-sectional study was conducted in Sana'a city, Yemen, from April 2012 to August 2012. This study is part of a bigger study. There were 16 large hospitals distributed in Sana'a city that had emergency department services (8 public and 8 private hospitals). Six hospitals were selected randomly from those 16 hospitals in the city (3 public and 3 private hospitals). Then, convenient samples of participants from each hospital were included in this study. Targeted women were those who attended the emergency department with their children in each hospital to ensure that all subjects were either married or previously married (widowed or divorced). Single women were excluded from this study. Six hundred and ninety-two women participated in this study. To ensure the diversity of the sample, data was collected during the whole day.

### 2.2. Ethical Issues

Our study protocol was approved by the National University of Malaysia's Ethics Committee in 2011, the Ministry of Health in Yemen, and by respective authorities of the selected hospitals where interviews took place. A brief explanation of the study's objectives was given to all respondents. Confidentiality was assured and oral consent was obtained since some subjects were illiterate.

### 2.3. Study Instruments

Women were interviewed “face to face” by the researcher and trained assistants using a structured questionnaire specifically developed for this study. From each hospital, two nurses with at least 2 years of experience were trained to conduct the interview and to fill up the questionnaire. The survey focused on four sociodemographic areas of concern: family, home, tradition, and “other” habituating factors. The questionnaire was designed for the purpose of this study. It was validated by using content and expert validation for both Arabic and English versions, before it was piloted by an initial cadre of thirty participants. The questionnaire was in Arabic, the official language of Yemen.

The dependent variable was khat chewing by women. Women were considered “khat chewers” if they chewed khat at least once weekly during the past six months. Those who never chewed khat and those who chewed khat less than once weekly in the past six months were considered “non-khat chewers.” In addition, family social gathering was defined as any gathering of at least 2 families at home or in social halls for more than two hours per visit.

### 2.4. Statistical Analysis

Data was analysed using the SPSS (Version 20). Descriptive statistics were conducted for all variables. Bivariate analysis was performed to obtain the unadjusted odds ratio (OR) and the 95% confidence interval (95% CI). Multiple backward stepwise regression (LR method) was used to specifically determine variables that were associated with khat chewing. Variables significantly associated with khat chewing in the bivariate analysis were entered into the multiple logistic regression analysis to obtain the adjusted OR.

## 3. Results

### 3.1. Descriptive and General Characteristics of Related Factors

Seven hundred and eight adult Yemeni women were recruited in this study. Six hundred and ninety-two completed questionnaires were analysed (97.7%). This study found that 205 women (29.6%) were khat chewers. Mean age (±SD) was 26.97 years (±5.9) and the age ranged from 17 to 42 years. In Table 1, the majority were married (82.9%), had secondary education (43.5%), and were unemployed. Most of the respondents had a total family income of YR ≤ 55,000 per month. Most of them lived in rented houses (63.9%), had a family of seven or more members (52.0%), and had more than three children (64.9%). Half of the women practiced family social gatherings with three times or less per week (50.6%). The majority of their husbands were khat chewers (53.6%) (Table 1).

### 3.2. Factors Associated with Chewing Khat among Women in Bivariate and Multivariate Analysis

Table 2 shows that khat chewing among women in bivariate analysis was significantly associated with age of women (*P* = 0.007), marital status (*P* = 0.005), and higher income (*P* = 0.003) (Table 2).

Khat chewing was also associated with family size (*P* = 0.017), large house (*P* = 0.040), frequent family social gatherings per week (*P* = 0.015), and chewing khat by husband (*P* < 0.001). Other factors were not significantly associated with chewing khat (*P* > 0.05) (Table 3).

In multivariate analysis, factors associated with chewing khat were khat chewing by husbands (OR = 1.8; CI 95%: 1.26–2.53, *P* = 0.001), being married (OR = 2.0; 95% CI: 1.20–3.37, *P* = 0.009), frequent social family gatherings per week (OR = 1.5; 95% CI: 1.06–2.10, *P* = 0.022), high family income (OR = 1.6; 95% CI: 1.12–2.21, *P* = 0.010), more than three rooms in the house (OR = 1.6; 95% CI: 1.16–2.31, *P* = 0.005), and age of women (OR = 0.6; 95% CI: 0.44–0.92, *P* = 0.015). Having chewing khat by husband was the most important factor in the model. If husbands chewed khat, wives were twice likely to chew khat compared to those whose husbands did not. This model was stable and statistically significant and fit the data (Hosmer and Lemeshow test; *P* > 0.05) (Table 4).

## 4. Discussion

This is the first study that explored khat chewing among Yemeni women with a specific focus on the role of the family and husbands. Previous studies reported that khat chewing was associated with age, gender, residence, and occupation [27]. The literature reported that the majority of khat chewers are between the age of 25–34 years for both sexes [28]. This study confirmed this finding as most khat chewers identified were between 26 and 35 years of age. Also it was discovered that married women were more likely to chew khat compared to divorced and widowed women. This might be caused by husbands who make khat more available while encouraging their wives to share the habit [29]. However, further qualitative research is required to clearly explain the role of husbands in the matter.

Previous studies reported that Yemeni khat chewers have higher unemployment rates and had lower levels of education, with their majority living in deprived areas with diminished levels of social interaction [9]. However, this study demonstrated that the level of education was not significantly associated with khat chewing. Likewise, no association was found between khat chewing and employment status. These results were not surprising, however, because the customary time for chewing khat usually begins after working hours in the evening. Moreover, most of the women queried did not work (70.2%) and remained at home as caregivers. Hence, the probability of a relation between work and khat chewing was indistinct. Furthermore, the impact on working hours was minimal because some husbands chewed khat at night, even if they were working overtime.

Women with higher incomes in this study were more likely to chew khat compared to those with lower incomes. This is because families with lower incomes may not have the mean to buy khat in lieu of family expenses. It was also found that khat chewing was higher among women with larger families and more rooms in homes compared to those with smaller families and smaller homes with fewer rooms. Large family size appears to increase the likelihood of family members who chew khat which possibly encourages other family members in the family chewing khat. A higher number of rooms in the house may also facilitate the allocation of a specific room for khat chewing in Yemeni society, one that is especially set aside to entertain guests and enjoy a “khat session.” We found that a higher number of rooms in the family domicile were significantly associated with khat chewing, whether the house was rented or owned.

This study also confirmed that a higher incidence of family gatherings was associated with khat chewing. Also it was demonstrated that khat chewing by husbands had a noticeable effect of nearly doubling the khat chewing by the women we surveyed, indicating that Yemeni women were influenced by the behaviour of their husbands and tended to share the habit. A study by Zeleke et al. in 2013 found that if one member of the family is khat chewer, this may influence khat chewing among other members in the family [28].

This study was limited by its cross-sectional design that cannot prove the causal relationship between variables. The participants also were enrolled from hospitals in a single city only. Hence, this study was undertaken at hospitals deemed the only practical locations where social barriers were somewhat eased. In addition, a household survey was impossible at the time of the study due to dangerous political disturbances at the time of data collection. Chewing khat before marriage was not investigated.

## 5. Conclusions

About one-third of the women participating in this study were khat chewers. This study also demonstrated that husbands and family venues clearly play significant roles in khat chewing by Yemeni women. Other factors were age, being currently married, higher family income, large houses, and frequent family gatherings. Urgent action is necessary to be done by the local authorities and the NGOs to control khat cultivation and its chewing. Creating awareness and increasing knowledge on the harmful effects of khat chewing are recommended. A particular attention should be given to women and the new generations. Further longitudinal research with bigger and representative sample size is required.

## Figures and Tables

**Table 1:** Sociodemographics, socioeconomic, and family context of the respondents.

Variables	Total
	*n* (%)
*Sociodemographic and socioeconomic factors *	
Women age	
≤25 years	280 (40.5)
26–35 years	336 (48.6)
>35 years	76 (11.0)
Marital status	
Previously married	118 (17.1)
Currently married	574 (82.9)
Husband age	
≤25 years	90 (13.0)
26–35 years	357 (51.6)
≥35 years	245 (35.4)
Husband education	
Postgraduate	150 (21.7)
Secondary	337 (48.7)
Primary	106 (15.3)
Illiterate	59 (8.5)
Women education	
Postgraduate	57 (8.2)
Secondary	301 (43.5)
Primary	171 (24.7)
Illiterate	148 (21.4)
Husband occupation	
Not working	133 (19.2)
Working	559 (80.8)
Husband working hrs/day	
>6 hours	547 (79.0)
≤6 hours	145 (21.0)
Women occupation	
Not working	486 (70.2)
Working	206 (29.8)
Women working hrs/day	
>6 hours	614 (88.7)
≤6 hours	78 (11.3)
Family income/month YR*	
≤55,000	365 (52.7)
>55,000	327 (47.3)

*Family context factors *	
House type	
Tenant	442 (63.9)
Owner	250 (36.1)
Size of the house	
≤3 rooms	349 (50.4)
>3 rooms	343 (49.6)
Family size	
<6 members	360 (52.0)
≥6 members	332 (48.0)
Number of children	
>3 children	449 (64.9)
≤3 children	243 (35.1)
Husbands chewed khat	
No	321 (46.4)
Yes	371 (53.6)
Frequency of chewing khat by husbands/week (*n* = 383)	
<4 times	174 (25.1)
≥4 times	197 (28.5)
Family social gatherings/week	
≤3 times	350 (50.6)
>3 times	342 (49.4)

*YR: Yemeni Riyal (the current currency unit).

**Table 2:** Association between sociodemographic and economic factors and khat chewing among Yemeni women (*n* = 692).

Variables	Khat chewer	Non-khat chewer	Crude OR (95% CI)	*P* value
	205 (29.6%)	487 (70.4%)		
Women age				
≤25 years	97 (34.6)	183 (65.4)	1	
26–35 years	83 (24.7)	253 (75.3)	1.6 (1.14–2.29)	**0.007**
>35 years	25 (32.9)	51 (67.1)	1.1 (0.63–1.85)	0.776
Marital status				
Previously married	22 (18.6)	96 (81.4)	1	
Currently married	183 (31.9)	391 (68.1)	2.0 (1.24–3.35)	**0.005**
Husband age				
≤25 years	31 (34.4)	59 (65.6)	1	
26–35 years	103 (28.9)	254 (71.1)	1.3 (0.79–2.12)	0.301
≥35 years	71 (29.0)	174 (71.0)	1.3 (0.77–2.15)	0.336
Husband education				
Postgraduate	54 (28.4)	136 (71.6)	1	
Secondary	93 (27.6)	244 (72.4)	0.9 (0.66–1.43)	0.839
Primary	34 (32.1)	72 (67.9)	1.2 (0.71–1.99)	0.510
Illiterate	24 (40.7)	35 (59.3)	1.7 (0.94–3.17)	0.078
Women education				
Postgraduate	23 (31.9)	49 (68.1)	1	
Secondary	75 (24.9)	226 (75.1)	0.7 (0.40–1.24)	0.225
Primary	63 (36.8)	108 (63.2)	1.2 (0.69–2.23)	0.466
Illiterate	44 (29.7)	104 (70.3)	0.9 (0.49–1.66)	0.738
Husband occupation				
Not working	40 (30.1)	93 (69.9)	1	
Working	165 (29.5)	394 (70.5)	0.9 (0.64–1.47)	0.899
Husband working hrs/day				
>6 hours	164 (30.0)	383 (70.0)	1	
≤6 hours	41 (28.3)	104 (71.7)	0.9 (0.61–1.38)	0.689
Women occupation				
Not working	150 (30.9)	336 (69.1)	1	
Working	55 (26.7)	151 (73.3)	0.8 (0.56–1.17)	0.273
Women working hrs/day				
>6 hours	187 (30.5)	427 (69.5)	1	
≤6 hours	18 (23.1)	60 (76.9)	0.7 (0.39–1.19)	0.181
Family income/month YR*				
≤55,000	90 (24.7)	275 (75.3)	1	
>55,000	115 (35.2)	212 (64.8)	1.7 (1.19–2.30)	**0.003**

*YR: Yemeni Riyal (the current currency unit in Yemen); simple logistic regression was used.

**Table 3:** Association between house, family context, and khat chewing among Yemeni women (*n* = 692).

Variables	Khat chewer	Non-khat chewer	OR (95% CI)	*P* value
	205 (29.6%)	487 (70.4%)		
House type				
Tenant	132 (29.9)	310 (70.1)	1	
Owner	73 (29.2)	177 (70.8)	1.0 (0.69–1.36)	0.85
Size of the house				
≤3 rooms	123 (35.2)	226 (64.8)	1	
>3 rooms	82 (23.9)	261 (76.1)	1.7 (1.24–2.41)	**0.001**
Family size				
<6 members	121 (33.6)	239 (66.4)	1	
≥6 members	84 (25.3)	248 (74.7)	1.5 (1.07–2.08)	**0.017**
Number of children				
>3 children	135 (30.1)	314 (69.9)	1	
≤3 children	70 (28.8)	173 (71.2)	0.9 (0.67–1.33)	0.729
Husbands chewed khat				
No	71 (22.1)	250 (77.9)	1	
Yes	134 (36.1)	237 (63.9)	2.0 (1.42–2.79)	**<0.001**
Frequency of chewing khat by husbands/week (*n* = 383)				
<4 times	56 (32.2)	118 (67.8)	1	
≥4 times	78 (39.6)	119 (60.4)	0.7 (0.47–1.11)	0.139
Family social gatherings/week				
≤3 times	89 (25.4)	261 (74.6)	1	
>3 times	116 (33.9)	226 (66.1)	1.5 (1.08–2.09)	**0.015**

Simple logistic regression was used.

**Table 4:** Factors associated with chewing khat among women in multivariate analysis (*n* = 692).

Variables	Category	*B*	S.E	*P* value	OR (95% CI)
Age of women/yrs	26–35	−0.45	0.19	0.015	0.6 ( 0.44–0.92)
	>35	0.07	0.29	0.808	1.1 (0.60–1.91)
	≤25				1
Marital status	Currently married	0.69	0.26	0.009	2.0 (1.20–3.37)
	Previously married				1
Husband chewed chewing khat	Yes	0.58	0.18	0.001	1.8 (1.26–2.53)
	No				1
Frequency of social family gathering/week	>3 times	0.40	0.18	0.022	1.5 (1.06–2.10)
	>3 times				1
Family income/month (YR)	>55,000 YR	0.45	0.17	0.010	1.6 ( 1.12–2.21)
	≤55,000 YR				1
Size of the house	>3 rooms	0.49	0.18	0.005	1.6 (1.16–2.31)
	≤3 rooms				1
Constant		−2.27	0.34	0.000	

Multiple logistic regression was used, and backward LR was used.

## Abbreviations

YR: Yemeni Riyal (the current Yemeni currency unit)
CI: Confidence interval
OR: Odds ratio
SD: Standard deviation
*n*: Number of all respondents
B: Unstandardized coefficients
SE: Standard error
*P* value: Level of significance.
